# Supplementary material for: Time‑resolved multi-omic analysis of paclitaxel exposure in human iPSC‑derived sensory neurons unveils mechanisms of chemotherapy‑induced peripheral neuropathy
Source: Cell Death Dis. 2026 Feb 10;17(1):211. doi: 10.1038/s41419-026-08445-2 (PMC12921266; doi:10.1038/s41419-026-08445-2)
Supplement: Supplementary file 15 — Dataset 1. [file 41419_2026_8445_MOESM15_ESM.zip › CDD_Open_Data/04_RNA_Seq/Timeline_panel_plots_etc_2023-01-23_1609.zip!/Panel_Plots_etc/Evidence_Plots/post5d/evidence_plot_standard contrasts_msigdb_reactome_5d_PTX_vs_DMSO_ID6_M838_REACTOME_UNBLOCKING_OF_NMDA_RECEPTORS_GLUTAMATE_BINDING_AND_ACTIVATION.pdf]

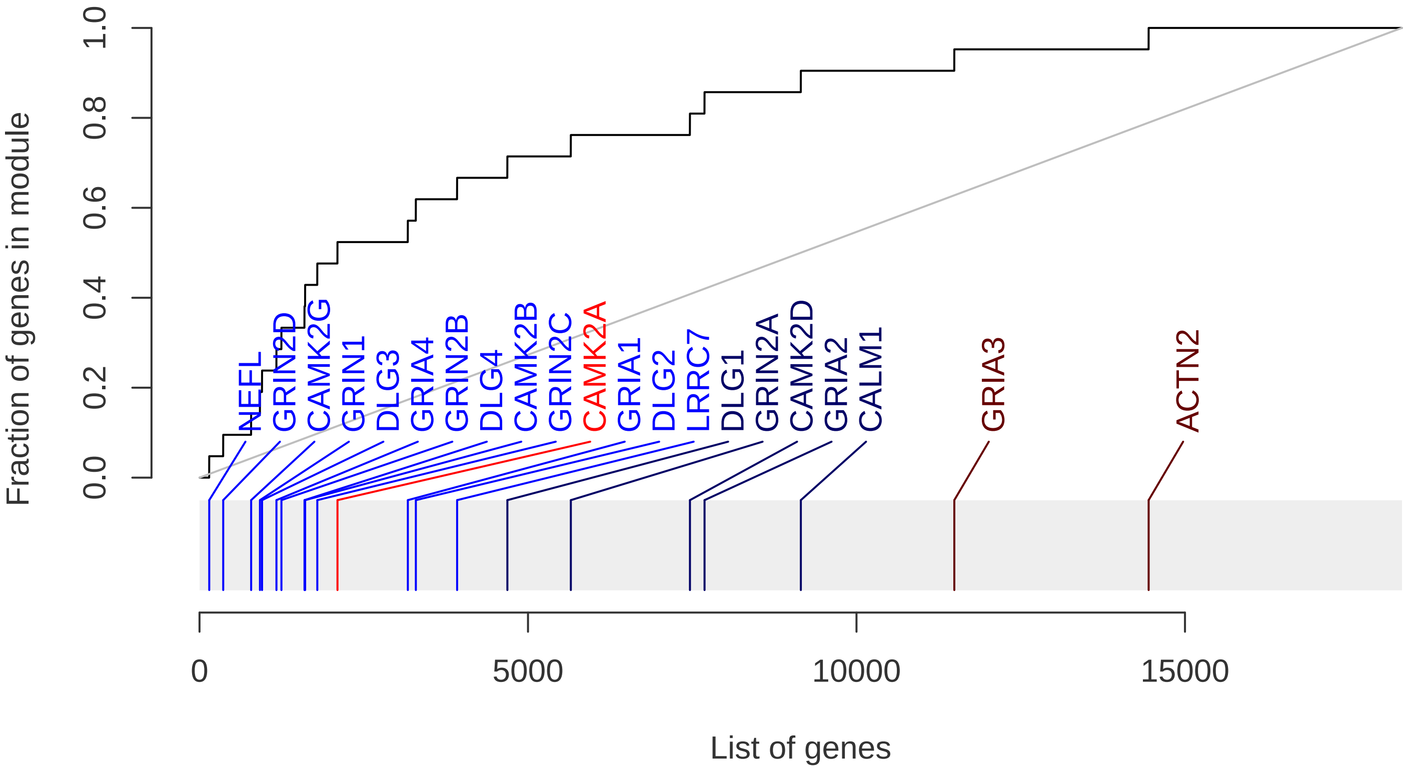

Fraction of genes in module
0.0
0.2
0.4
0.6
0.8
1.0
NEFL
GRIN2D
CAMK2G
GRIN1
DLG3
GRIA4
GRIN2B
DLG4
CAMK2B
GRIN2C
CAMK2A
GRIA1
DLG2
LRRC7
DLG1
GRIN2A
CAMK2D
GRIA2
CALM1
GRIA3
ACTN2
0
5000
10000
15000
List of genes
